# Patterns of smartphone usage associated with depressive symptoms in nursing students

**DOI:** 10.3389/fpsyt.2023.1136126

**Published:** 2023-08-03

**Authors:** Yajuan Yang, Mats Granlund, Fangbiao Tao, Shuman Tao, Liwei Zou, Xiaoyan Wu, Jingfang Hong, Karin Enskär

**Affiliations:** ^1^School of Nursing, Anhui Medical University, Hefei, China; ^2^CHILD, School of Health and Welfare, Jönköping University, Jönköping, Sweden; ^3^Department of Maternal, Child and Adolescent Health, School of Public Health, Anhui Medical University, Hefei, China; ^4^MOE Key Laboratory of Population Health Across Life Cycle, Hefei, China; ^5^Department of Women's and Children's Health, Uppsala University, Uppsala, Sweden

**Keywords:** problematic smartphone use, depressive symptoms, smartphone usage pattern, principal component analysis, students

## Abstract

**Introduction:**

Rather than focusing on the activities that the smartphone has been used for, the existing literature frequently focuses on the association between problematic use of smartphone independent of the content of use (self-reported) and depressive symptoms in youth. This study aims to explore patterns of smartphone usage and the association with depressive symptoms in nursing students.

**Methods:**

This cross-sectional study of nursing freshmen (*n* = 1, 716) was conducted between October and November 2018. Participants were recruited from three Chinese public medical universities using stratified cluster sampling. Self-rated frequency of 12 different smartphone activities over the preceding week was evaluated. Depressive symptoms were assessed by using the Patient Health Questionnaire-9 (PHQ-9).

**Results:**

Of the 1,716 students recruited, 1,424 (83.0%) were girls, and the mean [SD] age was 18.90 [1.39] years. Using principal component analysis (PCA), two typical usage patterns were indicated. The “entertainment pattern” factor included a high frequency of streaming images or videos, searching for information, chatting online, online shopping, downloading, reading online, checking social media sites, taking pictures or videos, and playing games. The “communication pattern” had a high frequency of emailing, texting, and calling. Using logistic regression models, the association between smartphone usage patterns and depressive symptoms was tested. The “communication pattern” was significantly associated with a 53% increase in the odds of moderate and above depressive symptoms (AOR = 1.529; 95% CI = 1.286–1.818; *p* < 0.001), controlling for a set of socio-demographic and smartphone use covariates.

**Discussion:**

This study provides insights into how the patterns of smartphone usage are associated with the severity of depressive symptoms in nursing students. It indicates that it may primarily be how we use our smartphones rather than how much we use them that poses a risk for depression.

## 1. Introduction

The World Health Organization (1) reports a concern about the potential negative health impacts of cell phones on children and adolescents due to their widespread global appeal. Therefore, one of the top priorities for research is to examine the effects of the high use of smartphones. By December 2020 in China, nearly a billion Internet users make up the world's largest digital society. Compared to TV, computer, laptop, and other devices, smartphones were the most popular access to the Internet (99.6%), according to the newest report from China Internet Network Information Center (2).

Smartphones create opportunities for learning, development, and personal growth in adolescents and young adults. However, there is some evidence that some individuals develop a pattern of smartphone use that is problematic due to the instantaneous nature, anonymity, broad reach, and lack of regulation of the content of smartphones, as well as the immature self-regulation in adolescence (3). This public health issue has been termed “smartphone addiction” (4), “smartphone dependency” (5), “proneness to smartphone addiction” (6), “excessive smartphone use” (7), or “smartphone overuse (8). Recently, researchers have avoided the use of the term “addiction” and instead used “problematic smartphone use” (PSU) to conceptualize the adversity associated with excessive smartphone use (9). The framework of PSU is characterized by symptoms such as withdrawal (negative effect when separated from one's smartphone), craving (attempts to increase smartphone use), and physical and mental influence (problems in daily life/school and/or health) (10). However, inconsistent estimates of the prevalence of PSU are reported due to various definitions and criteria used (11–14). A systematic review of the association between problematic smartphone usage and mental health outcomes among children and young people (15) found that the median prevalence of PSU in children and young people was 23.3% (14.0–31.2%), which measured by a range of scales, such as the Mobile Phone Problematic Use Scale (MPPUS) or the Smartphone Addiction Scale (SAS).

In the healthcare sector, women account for 70%, with nurses (including midwives) accounting for the majority (8). A questionnaire survey of 3,311 nurses in 40 hospitals in eight provinces and cities in China revealed that nurses who felt depressive symptoms were above 86%. This finding is in line with the results of a survey on the physical and mental conditions of medical staff in more than 80 hospitals of all levels in 10 provinces and cities in China in 2007 (16). This not only affects their careers but also has an impact on patient safety and quality of care (17). It can even affect the next generation by damaging the reproductive system (18). Given that changes in nurses' mental health status are a gradual process, long-term monitoring and early prevention from student days are particularly important.

Mental health problems associated with PSU are reported across cultures, from Europe (14), America (19), and Asia (10). A systematic review conducted by Elhai et al. demonstrated that the severity of depression was consistently associated with PSU, showing at least medium effects (20). However, empirical research on the relationship between smartphone function and depression depressive symptoms is not thorough.

As technology advances, smartphones offer a range of functions, from communication to entertainment. Nowadays, smartphone technology can be characterized by uses such as basic applications (e.g., instant messaging and search engines), business transactions (e.g., online shopping and online payment), entertainment online (e.g., online games and online video), and public service (e.g., online car-hailing and online medical service) in China (2). A systematic review showed that all domains of social media (time spent, activity, investment, and addiction) correlated with depression (21). However, there are some different voices. The results of a cross-cultural study showed that the most popular uses were ranked the same in all three countries: (1) texting, (2) reading social content, and (3) browsing the Internet. Regarding depression scores, in the United States, texting was a contributing factor. In Spain, texting was a facilitator, and posting social content was a protective factor. In Colombia, playing games was a protective factor (22). Another study (23) found that depressive symptoms were inversely associated with the social use of smartphones. In addition, some researchers explore the gender differences in smartphone usage and their relationship with depressive symptoms (24, 25). These inconsistent findings may occur depending on the different cultures, ages, majors, gender, and so on.

In order to develop tailored intervention programs in future, more knowledge is needed about if certain patterns of smartphone usage are associated with depressive symptoms. Based on Chinese culture, we sought to characterize smartphone usage patterns among nursing students and to evaluate the associations between these patterns and depressive symptoms.

## 2. Materials and methods

### 2.1. Participants and recruitment

A sample of 1,954 nursing freshmen was recruited from three medical universities located in Anhui Province between October and November 2018. First, three universities were selected by convenient sampling. Then, freshmen from nursing school were selected based on stratified cluster sampling. All participants were approached in class in their first semester as the study subjects. After the deduction of attrition due to declining participation and invalid questionnaires, 1,716 valid questionnaires were recovered with a recovery rate of 87.8%. The average age of the subjects was 18.90 ± 1.39 years old (16–26 years), including 292 boys (17.0%) and 1,424 girls (83.0%).

Ethical approval (No. 20170219) was obtained from the Ethical Committees of Anhui Medical University before data collection. Informed written consent was provided by all participants before participation.

### 2.2. Procedures

Participants were assessed in their classroom after class by four trained investigators. They completed the paperless questionnaire with the WeChat App on their phones. Participants were informed that it was voluntary to participate and that they were free not to respond to questions. All questionnaires were without personal information, but each respondent was assigned a code. With the help of a code key stored separately from the data, the researchers could match a person to questionnaires, a prerequisite for the following data collection.

### 2.3. Measures

#### 2.3.1. Depressive symptoms

The Patient Health Questionnaire-9 (PHQ-9) is a self-assessed instrument used for detecting depressive symptoms and evaluating the severity of depressive symptoms in a primary care setting (26). It was widely used in the depression assessment of different diseases in Chinese comprehensive or community health centers, with good credit validity (12). It is composed of nine items inquiring about the level of each participant's depressive symptoms. Each item is scored “0” (not at all) to “3” (nearly every day), with the highest total score of 27. Depressive symptoms were classified as minimal (score, 0–4), mild (score, 5–9), moderate (score, 10–14), moderately severe (score, 15–19), and severe (score, 20–27). Cronbach's alpha coefficient was 0.89 in the primary care study (27) and 0.91 in this study.

#### 2.3.2. Smartphone usage

It was measured with an instrument adapted from Ra et al. (28) for use with Chinese university students. The original questionnaire was developed to indicate how frequently users engaged in media activities in the past week, which we adapted to how frequently participants engaged in smartphone-using activities (e.g., streaming images or videos/chatting/searching information/reading/emailing/calling/shopping/taking pictures or videos/checking social media sites/downloading/playing games/texting). The frequency of use was reported as never, 1–2 times/week, 1–2 times/day, or multiple (i.e., more than three) times/day. The results of the pretest showed its Cronbach's alpha coefficient was 0.79.

#### 2.3.3. Covariates

Several sociodemographic variables were adjusted that may affect smartphone usage (29), which in turn could affect depression (30). These include age (years), gender (male/female), number of friends (< 3/3–5/>5), household income (low/medium/high), critical negative life events in the last year (changes in the family [yes/no]/broken up in love [yes/no]/hospitalizing [yes/no]), and family depression history (yes/no).

The Chinese version of the Pittsburgh Sleep Quality Index (PSQI) was used to assess the quality of sleep of university students in the last month (31). The PSQI consists of 19 entries covering sleep quality, time to fall asleep, sleep duration, sleep efficiency, sleep disorders, hypnotic medication use, and daytime dysfunction. Each dimension is scored from 0 to 3. The total score ranges from 0 to 21, with a score of ≥8 indicating poor sleep quality (32). Cronbach's alpha coefficient was 0.87 in the present study.

Physical activity was assessed using the International Physical Activity Scale (IPAQ) (33). Physical activity was divided into three categories walking, moderate intensity (lifting lighter objects, swimming, cycling, etc.), and high intensity (lifting heavier objects and running, etc.). and their metabolic equivalent (MET) were assigned to 3.3, 4.0, and 8.0. The total amount of MET is equal to the sum of three intensity METs (34). The scoring criteria are as follows: High physical activity was defined as a total of ≥3 days of all types of high-intensity physical activity and an overall weekly physical activity level of ≥1,500 MET, or a total of ≥7 days of all three types of high-intensity physical activity and an overall weekly physical activity level of ≥3,000 MET. Moderate physical activity is defined as meeting the criteria of ≥3 days of all types of high-intensity physical activity for at least 20 min per day combined or ≥5 days of all types of moderate-intensity/or walking activity for at least 30 min per day combined, or ≥5 days of all three types of high-intensity physical activity combined and a total weekly physical activity level of ≥600 MET. Low physical activity was defined as not reporting any activity or reporting some activity but not meeting the above criteria for the medium and high groups (35). Cronbach's alpha coefficient was 0.89.

Smartphone use covariates included smartphone use duration and PSU. First, we asked participants to estimate the total time, i.e., duration (open-ended boxes for hours and minutes) spent on a smartphone for personal use per day. The total time was converted into minutes for analysis. Then, Mobile Phone Dependency self-rating Questionnaire for Adolescent Mobile Phone Use (SQAPMPU) (10) was used to indicate if they use the smartphone in a problematic manner (see Appendix A). This questionnaire consists of 13 items assessing three dimensions: (1) withdrawal symptoms (e.g., When I attempt to spend less time on or stop using my mobile phone, I feel upset or irritated/I become irritable if I have to switch off my mobile phone for meetings, dinner engagements, or at the movies/when out of range for some time, I become preoccupied with the thought of missing a call), (2) physical and mental influence (e.g., I lose sleep due to the time I spend on my mobile phone/my leisure activities are reduced due to the time I spend on my mobile phone/my productivity has decreased as a direct result of the time I spend on the mobile phone), and (3) craving (e.g., I can never spend enough time on my mobile phone/I need to spend more time on my mobile phone to be satisfied/I have frequent dreams about the mobile phone.). Responses are provided on a 5-point Likert scale from 1 (not true at all) to 5 (extremely true). The validity and reliability of the SQAPMPU have been previously examined (36). The total score ranges from 13 to 65, with the 75th percentile used as the cutoff point for exhibiting problematic use. Primary scores were recoded as “no” (<P75) or “yes” (≥P75) (36). Therefore, scores ≥26 were defined as PSU, and Cronbach's coefficient was 0.92 in the present study.

### 2.4. Data analysis

All analyses were performed with SPSS version 25.0. Absolute frequencies and percentages or means and standard deviations (SDs) were computed for categorical and continuous variables, respectively. Chi-square tests or Kruskal–Wallis *H*-tests were used for three-group (minimal/mild/moderate to severe depression) comparisons, as appropriate. A significance level of *p* < 0.05 was adopted for these analyses. Principal-component analysis (PCA) was performed to extract the participants' smartphone usage patterns using 12 activities to create factors. A varimax rotation was used for the interpretability of the factors that were analyzed. The number of factors retained was based on the screen test (37), eigenvalues >1, the proportion of variance explained by each factor and by all factors retained, and interpretability (38). The patterns of smartphone usage were named according to the highest factor loading for mobile phone activities. Each pattern's factor score was prepared for further analysis. We then used a multinominal logistic regression model (Model: enter) to analyze the correlation between the severity of depressive symptoms and smartphone usage patterns. At the same time, number of friends, household income, academic performance, sleeping quality, physical activity level, PSU, being hospitalized in the past 1 year, examination failure in the past 1 year, break up/in love in the past 1 year, and smartphone use duration were selected for statistically significant differences in the univariate analysis and adjusted to obtain the OR and 95% CI. Any *P*-values < 0.05 were considered statistically significant.

## 3. Results

### 3.1. Participants

Among 1,716 included participants, the average age was 18.9 years (SD = 1.39), including 292 boys (17.0%) and 1,424 girls (83.0%) (Table 1). The majority (61.7%) of the participants were from rural areas. Notably, 24.7% were from a family with a low yearly household income, and 70.3% were from a medium household income.

**Table 1:** Whole sample characteristics and associations with level of depression severity (*N* = 1,716).

**Independent variable and covariates**		**Whole sample**	**Level of depression severity**			***F/χ^2^***	***P***
			**Minimal**	**Mild**	**Moderate and above**		
Age (mean ± SD)		18.90 ± 1.39	18.90 ± 1.42	18.88 ± 1.37	18.96 ± 1.27	1.579	0.454
Gender	Female	1,424 (83.0)	888 (62.4)	417 (28.3)	119 (8.3)	0.612	0.736
	Male	292 (17.0%)	187 (64.0)	79 (27.1)	26 (8.9)		
Residence	Rural	1,060 (61.8)	658 (62.1)	308 (29.1)	94 (8.8)	0.740	0.691
	Urban	656 (38.2)	417 (63.6)	188 (28.7)	51 (7.7)		
Number of friends	< 3	212 (12.4)	110 (51.9)	72 (34.0)	30 (14.1)	29.366	0.000
	3~5	583 (34.0)	342 (58.7)	184 (31.6)	57 (9.7)		
	>5	921 (53.6)	623 (67.6)	240 (26.1)	58 (6.3)		
Household income	Low	424 (24.7)	230 (54.3)	148 (34.9)	46 (10.8)	17.600	0.001
	Medium	1,206 (70.3)	786 (65.2)	327 (27.1)	93 (7.7)		
	High	86 (5.0)	59 (68.6)	21 (24.4)	6 (7.0)		
Academic performance	Poor	267 (15.6)	138 (51.7)	80 (30.0)	49 (18.3)	50.054	0.000
	Moderate	1,255 (73.1)	797 (63.5)	376 (30.0)	82 (6.5)		
	Excellent	194 (11.3)	140 (72.2)	40 (20.6)	14 (7.2)		
Sleeping quality	Good	1,538 (89.6)	1,040 (67.6)	410 (26.7)	88 (5.7)	215.058	0.000
	Bad	178 (10.4)	35 (19.7)	86 (48.3)	57 (32.0)		
Physical activity level	Low	538 (31.4)	311 (57.8)	170 (31.6)	57 (10.6)	11.048	0.026
	Medium	1,074 (62.6)	703 (65.5)	292 (27.2)	79 (7.3)		
	High	104 (6.0)	61 (58.7)	34 (32.7)	9 (8.6)		
Facing big family trouble in the past 1 year	Yes	214 (12.5)	119 (55.6)	73 (34.1)	22 (10.3)	5.197	0.074
	No	1,502 (87.5)	956 (63.6)	423 (28.2)	123 (8.2)		
Be hospitalized in the past 1 year	Yes	166 (9.7)	85 (51.2)	61 (36.7)	20 (12.1)	10.567	0.005
	No	1,550 (90.3)	990 (63.9)	435 (28.1)	125 (8.0)		
Examination failure in the past 1 year	Yes	1,180 (68.8)	696 (59.0)	372 (31.5)	112 (9.5)	21.919	0.000
	No	536 (31.2)	379 (70.7)	124 (23.1)	33 (6.2)		
Break up/in love in the past 1 year	Yes	346 (20.2)	187 (54.1)	118 (34.1)	41 (11.8)	15.098	0.001
	No	1,370 (79.8)	888 (64.8)	378 (27.6)	104 (7.6)		
Family history of depression	Yes	47 (2.7)	24 (51.1)	18 (38.3)	5 (10.6)	2.784	0.249
	No	1,669 (97.3)	1,051 (63.0)	478 (28.6)	140 (8.4)		
Smartphone use duration	< 2 h/days	66 (3.8)	52 (78.8)	9 (13.6)	5 (7.6)	19.862	0.003
	4~ h/days	636 (37.1)	388 (61.0)	193 (30.3)	55 (8.7)		
	≥6 h/days	614 (35.8)	363 (59.1)	188 (30.6)	63 (10.3)		
PSU	No	1,247 (72.7)	911 (73.1)	301 (24.1)	35 (2.8)	286.734	0.000
	Yes	469 (27.3)	164 (35.0)	195 (41.6)	110 (23.4)		

### 3.2. Patterns of smartphone usage

Two smartphone use patterns were identified by the principal component analysis (Table 2), including the “entertainment pattern” and the “communication pattern.” The “entertainment pattern” cluster was characterized by high loadings of items related to streaming images or videos, searching for information in social media, chatting online, online shopping, downloading, reading online, checking social media sites, taking pictures or videos, and laying games. The “communication pattern” was heavily loaded with items related to emailing, texting, and calling. Together, the two smartphone usage patterns explained 44.79% of the total variation. Analysis of the steep slope map as well as interpretation ability judgment suggested that extraction of the first two principal components had both statistical and theoretical relevance.

**Table 2:** Factor structure and loadings of smartphone usage (past 1 week)^a^.

**Smartphone usage**	**Loading**	
	**Factor 1**	**Factor 2**
Streaming images or videos (including TV or movies)	**0.766**	−0.053
Searching information	**0.692**	0.042
Chatting online (e.g., QQ or Wechat)	**0.665**	−0.097
Online shopping (e.g., Taobao or Jingdong)	**0.605**	0.283
Reading online (e.g., articles, news, or books)	**0.584**	0.150
Taking pictures or videos	**0.583**	0.198
Checking social media sites (e.g., blogs)	**0.579**	0.154
Downloading	**0.570**	0.391
Playing games	**0.400**	0.111
E-mailing	−0.055	**0.882**
Texting	0.098	**0.799**
Calling	0.237	**0.521**
% Explained variance	28.695	16.092

a Principal component analysis (with a varimax rotation).

KMO = 0.853, *P* < 0.001.

Bold: correlation coefficient ≥0.4.

### 3.3. Association between patterns of smartphone usage and severity of depressive symptoms

Table 3 shows the results from the multiple logistic regression models. The “entertainment pattern” and “communication pattern” patterns were both significantly associated with a 17% (OR = 1.167; 95% CI = 1.047–1.300; *p* < 0.01) and 13% (OR = 1.128; 95% CI = 1.009–1.262; *p* < 0.01), respectively, increase in odds of mild depressive symptoms. Only the “communication pattern” was related to a 77% increase in the odds of moderate and above depressive symptoms (OR = 1.774; 95% CI = 1.543–2.041; *p* < 0.001). To account for the confounding effect of other variables, models were adjusted for number of friends, household income, academic performance, sleeping quality, physical activity level, being hospitalized in the past 1 year, examination failure in the past 1 year, break up/in love in the past 1 year, smartphone use duration, and PSU. Despite the slight attenuation of all the effects, significant associations between the “communication pattern” and moderate and above depressive symptoms remained (AOR = 1.529; 95% CI = 1.286–1.818; *p* < 0.001).

**Table 3:** Association between smartphone usage patterns and depression severity (*N* = 1,716).

**Smartphone usage patterns**	**Model**	**Depression severity**		
		***OR*** **[95% CI]**		
		**None**	**Mild**	**Moderate and above**
**Entertainment pattern**				
	Crude	Reference	1.167 (1.047–1.300)^b^	1.164 (0.975–1.389)
	Adjusted^a^	Reference	1.065 (0.939–1.209)	0.896 (0.716–1.122)
**Communication pattern**				
	Crude	Reference	1.128 (1.009–1.262)^b^	1.774 (1.543–2.041)^c^
	Adjusted^a^	Reference	1.082 (0.955–1.224)	1.529 (1.286–1.818)^c^

a Adjusted for the number of friends, household income, academic performance, sleeping quality, physical activity level, being hospitalized in the past 1 year, examination failure in the past 1 year, break up/in love in the past 1 year, smartphone use duration, PSU.

b Indicates significant level *P* < 0.01.

c Indicates significant level *P* < 0.001.

## 4. Discussion

In this cross-sectional study, we investigated the smartphone usage of 1,716 nursing students and revealed two smartphone usage patterns (“communication pattern” and “entertainment pattern”), which together explained 44.79% of the total variance. We also found that communication and entertainment pattern were deferentially associated with depressive symptoms.

Earlier studies indicated a distinction in smartphone usage between process-oriented and social-oriented uses (39, 40). Social usage includes social networking, messaging, phone calls, and maintaining social relationships, and process usage includes news consumption, entertainment, relaxation, and other primarily non-social purposes. However, first, due to cultural differences, different countries and different groups of people may have different habits of using smartphones. Today, young adults use it not only as a tool for interpersonal communication *via* voice or text but also for entertainment, relaxation, information, various applications, taking photos/videos, passing the time, and as a symbol of identity and status in China. In the present study, the “entertainment pattern” included not only the variety of relaxation activities (e.g., streaming images or videos, online shopping, taking pictures, or videos), but also the use of social media (e.g., chatting online and checking social media sites). The “communication pattern” was primarily focused on contacting people (e.g., emailing, texting, and calling). Due to the orthogonal transformation in PCA, the use of two patterns we identified was statistically independent of one another, which solves the problem of correlation and multicollinearity between independent variables and makes the subsequent regression model of the relationship between smartphone use and depressive symptoms more stable. Our study is also highly consistent with (41) findings that most adolescents use their smartphones to communicate with each other in their daily lives (e.g., social network service, making phone calls, sending messages, and emails) or to have fun (e.g., watching TV and playing games), but rarely use them to engage in academic activities (e.g., finding study materials), except for social network use.

Both “entertainment pattern” (OR = 1.17) and “communication pattern” (OR = 1.23) had a significant association with mild depressive symptoms. However, neither “entertainment pattern” nor “communication pattern” was a determinant since the association failed to reach the 0.05 level in the adjusted model. However, odds ratios for both smartphone usage patterns remained relatively unaffected by the adjustment, suggesting that they may put nursing students at a likely increased risk of developing depressive symptoms. On the other hand, the association between “communication pattern” and moderate and above depressive symptoms was significant compared to “entertainment pattern” and persisted after statistical adjustment for other relevant variables, but was attenuated after adjustment for relevant variables, including number of friends, household income, academic performance, sleeping quality, physical activity level, being hospitalized in the past 1 year, examination failure in the past 1 year, break up/in love in the past 1 year, smartphone use duration, and PSU. These findings suggest that the relationship between “communication pattern” and moderate and above depressive symptoms in nursing students is complex. It is recommended that the influence of these factors be considered in future research.

This is consistent with a Japanese study showing that depression was associated with text messaging as the primary means of communication, including short message service (SMS) communications and emails (42). The results of a recent cross-cultural study also showed that regarding depression scores, texting was a contributing factor in the United States. In Spain, texting was a contributing factor, while posting social content was a protective factor (22). One possible reason for this is related to concerns about relationship maintenance among text-dependent individuals and the presence of personality immaturity among this type of adolescent, such as poorer self-model of adult attachment and lower self-directedness, thus increased depression (43). Another explanation is that depressive symptoms are more common in freshmen than in the general population due to their recent departure from home and the interpersonal and academic pressures they face (44). They may use phone calls or text messages to release stress and seek support. In addition, people who are experiencing more depressive symptoms are more likely to engage in behavioral avoidance or social isolation by not using smartphones to alleviate their distress (45). Depressed users, therefore, may cease generating social media content (46) and focus on the communication use of the smartphone. Thus, only the communication pattern is related to moderate and above depressive symptoms, and the direction of the association might be reversed; that is, depressive symptoms lead to less use of entertainment function. However, this hypothesis has to be confirmed by longitudinal data.

Combined, these findings do not support the prevailing hypothesis that social media use correlates with moderate-to-severe depressive symptoms (21). This finding in the present study is consistent with recent research that active use of social media (i.e., social interaction, more frequent posting, liking, and commenting) can benefit overall mental health (40). This may be related to the Uses and Gratifications Theory [UGT; (47)]. Distinguishing from other theories, UGT emphasizes the active role of the audience in media selection and use. Based on this, UGT emphasizes and highlights the intrinsic psychological needs of individuals in terms of motivation and social needs to analyze the individual's choice and use of a particular medium (48). In summary, the relationship between smartphone use and depressive symptoms is complex, as these factors are highly interrelated. Future studies should consider a wider range of smartphone features (screen time, motivation to use, frequency of use, and passive and active use) to investigate the impact of smartphone activities on depressive symptoms.

Limitations include using a convenience sample of participants from nursing students, which is biased toward the female gender and limits the generalizability of findings to a wider population. Second, self-report measures were relied on in the present study, which may not be externally validated and introduce bias and inaccuracies in responses. In future, phone logs can be improved to measure common mobile phone usage activities and frequencies to conduct such studies. Third, there is a possible bias due to the interpolation between smartphone duration and type, i.e., the effect of media multitasking (MMT) (49), which may affect the accuracy of the results. In addition, the research questions were only explored at a single cross-section in time. Repeated measurement design is recommended for the future to objectively measure smartphone use and to provide a more fine-grained analysis of various characteristics of smartphone use.

## 5. Conclusion

The present study found that the communication pattern of smartphone usage was independently associated with higher levels of severity of depressive symptoms in nursing students. The result suggests that it primarily may be how students use smartphones, not how frequently they use phones, that is related to depressive symptoms. The type of use of smartphones, not only the frequency of use, should be considered in order to prevent depressive symptoms among nursing students. Whether depression elicits more texting or if the reveres association is seen has to be investigated in future research.

## Data availability statement

The original contributions presented in the study are included in the article/Supplementary material, further inquiries can be directed to the corresponding authors.

## Ethics statement

The studies involving human participants were reviewed and approved by Ethic Committees of Anhui Medical University. The patients/participants provided their written informed consent to participate in this study.

## Author contributions

Initial concept and design: FT, KE, YY, and XW. Data acquisition: YY and LZ. Statistical analyses: YY and MG. Contribution to the interpretation of the results: YY, JH, and MG. Preparation of the manuscript: YY. Preparation of tables and figures: ST. Revision of manuscript: MG, KE, and JH. All authors participated in the study design and provided academic contributions to the development of this manuscript and approved the final manuscript.
