# Supplementary material for: Patterns of smartphone usage associated with depressive symptoms in nursing students
Source: Front Psychiatry. 2023 Aug 3;14:1136126. doi: 10.3389/fpsyt.2023.1136126 (PMC10434770; doi:10.3389/fpsyt.2023.1136126)
Supplement: Supplementary file 1 [file Data_Sheet_1.docx]

Supplementary Material

Patterns of Smartphone Usage Associated with Depressive symptoms in Nursing Students

# Supplementary Figures and Tables

Appendix A

Self-rating Questionnaire for Adolescent Problematic Mobile Phone Use (SQAPMPU)

| **Dimensions** | **No.** | **Items** | **Not true at all** | **Slightly**  **true** | **Moderately true** | **Strongly true** | **Extremely true** |
| --- | --- | --- | --- | --- | --- | --- | --- |
| **Withdrawal symptoms** | 2 | When I attempt to spend less time on or stop using my mobile phone, I feel upset or irritated. | 1 | 2 | 3 | 4 | 5 |
|  | 4 | I become irritable if I have to switch off my mobile phone for meetings, dinner engagements, or at the movies. | 1 | 2 | 3 | 4 | 5 |
|  | 6 | When out of range for some time, I become preoccupied with the thought of missing a call. | 1 | 2 | 3 | 4 | 5 |
|  | 8 | I hear the phone ringing when it actually hasn’t which is called “ringxiety”, I always check my mobile phone [involuntarily](javascript:void(0);). | 1 | 2 | 3 | 4 | 5 |
|  | 11 | I feel anxious if I have not checked for messages or switched on my mobile phone for some time. | 1 | 2 | 3 | 4 | 5 |
|  | 13 | I feel lost without my mobile phone. | 1 | 2 | 3 | 4 | 5 |
| **Craving** | 1 | I can never spend enough time on my mobile phone. | 1 | 2 | 3 | 4 | 5 |
|  | 7 | I need to spend more time on my mobile phone to be satisfied. | 1 | 2 | 3 | 4 | 5 |
|  | 10 | I have frequent dreams about the mobile phone. | 1 | 2 | 3 | 4 | 5 |
| **Physical and mental health status** | 3 | I lose sleep due to the time I spend on my mobile phone. | 1 | 2 | 3 | 4 | 5 |
|  | 5 | There are times when I would rather use the mobile phone than deal with other more pressing issues. | 1 | 2 | 3 | 4 | 5 |
|  | 9 | My leisure activities are reduced due to the time I spend on my mobile phone. | 1 | 2 | 3 | 4 | 5 |
|  | 12 | My productivity has decreased as a direct result of the time I spend on the mobile phone. | 1 | 2 | 3 | 4 | 5 |
